# UMSARS Versus Laryngoscopy‐Based Assessment of Dysphagia

**DOI:** 10.1002/mdc3.13734

**Published:** 2023-04-12

**Authors:** Nadia El Fassi, Anne Pavy le Traon, Emmanuelle Mouchon, Olivier Rascol, Wassilios G. Meissner, Alexandra Foubert‐Saumier, Yohan Gallois, Samuel Tessier, Fabienne Ory‐Magne, Virginie A. Woisard, Margherita Fabbri

**Affiliations:** ^1^ Department of ENT Hospital of Larrey Toulouse France; ^2^ French Reference Center for Multiple System Atrophy, Neurology Department Toulouse university hospital and Institute of Metabolic and Cardiovascular Diseases INSERM UMR 1297 Toulouse France; ^3^ Clinical Investigation Center CIC1436, Department of Clinical Pharmacology and Neurosciences, Parkinson Expert Centre and NeuroToul Center of Excellence in Neurodegeneration (COEN) of Toulouse INSERM, University of Toulouse 3, CHU of Toulouse Toulouse France; ^4^ CHU Bordeaux, Service de Neurologie des Maladies Neurodégénératives, IMNc Bordeaux France; ^5^ Univ. Bordeaux, CNRS, IMN, UMR 5293 Bordeaux France; ^6^ Dept. Medicine University of Otago, Christchurch, and New Zealand Brain Research Institute Christchurch New Zealand; ^7^ Service de Pharmacologie Médicale et Clinique, Centre de Pharmacovigilance, Faculté de Médecine, de Pharmacoépidémiologie et d'Informations Sur Le Médicament CIC INSERM 1436, Centre Hospitalier Universitaire Toulouse France; ^8^ Department of Neurology University Hospital of Toulouse Toulouse France

**Keywords:** multiple system atrophy, dysphagia, UMSARS, ear nose and throat assessment

## Abstract

**Background:**

Multiple System Atrophy (MSA) dysphagia is routinely assessed by the Unified Multiple System Atrophy Rating Scale (UMSARS) part I‐item 2.

**Objective:**

To compare the UMSARS part I‐item 2 with an ear/nose/throat (ENT) expert physician assessment.

**Methods:**

We retrospectively analyzed the data of MSA patients who underwent an ENT assessment (nasofibroscopic and radioscopic exam) and an annual UMSARS assessment. Deglutition Handicap Index (DHI) and pulmonary/nutrition complications were collected.

**Results:**

Seventy‐five MSA patients were included. The ENT assessment revealed more severe dysphagia compared to the UMSARS part I‐item 2 score (*P* = 0.003). A higher proportion of patients with impaired protective mechanisms showed severe UMSARS‐based dysphagia (*P* = 0.005). Patients with choking and oral/pharyngeal transit defects and nutritional complications were equally distributed across UMSARS part I‐item 2 scores. Worse UMSARS part I‐item 2 scores had worse DHI scores.

**Conclusions:**

The UMSARS‐based assessment of dysphagia does not capture key aspects of pharyngo‐laryngeal dysfunction reflecting swallowing efficiency.

Multiple system atrophy (MSA) is an adult‐onset, progressive neurodegenerative disorder, clinically characterized by various combinations of autonomic failure, parkinsonism and ataxia.^1^, ^2^ Early and severe pharyngo‐laryngeal signs, including dysphagia and stridor, are peculiar signs of MSA, possibly presenting a source of major disability and a predictor of poor outcome.^3^, ^4^, ^5^ Dysphagia is frequent (31%–78%)^4^ and its presence within 3 years of disease motor symptom onset is listed among the supportive clinical features for MSA diagnosis.^6^

In clinical practice, swallowing problems are usually assessed by movement disorder specialists by means of the Unified Multiple System Atrophy Rating Scale (UMSARS) part I (item 2).^7^ The assessment of MSA patients by ear, nose, and throat (ENT) specialists is not systematically performed. It depends on clinical practice and is frequently reserved to patients with more severe swallowing disorders, dysphonia or inspiratory noises. Noteworthy, laryngoscopy as part of the ENT examination can inform on complete oro‐pharyngo‐laryngeal function.^8^

The severity of swallowing disorders can be defined according to two concepts. The first corresponds to the concept of “swallowing safety”; the ability to transfer the food bolus from the mouth to the stomach, without laryngeal penetration or inhalation in the lower airways. The second feature is “swallowing efficiency”; the ability to transfer the food bolus from the mouth to the stomach without oral/pharyngeal residue after swallowing. This second concept is not covered by the UMSARS “swallowing” item, although level 4 concerns the need for enteral nutrition.

The main objective of this study was to determine the correlation between the UMSARS part I item 2 score with an ENT assessment in the assessment of swallowing severity.

## Patients and Methods

### Study Protocol and Patient Criteria

We performed a retrospective, single‐center study on 301 MSA patients who were enrolled at the Toulouse site of the French MSA Reference Centre between January 2008 and March 2021. Clinical data were extracted from a health data hosting service (ORBIS). MSA patients from the Toulouse Centre are part of the French MSA cohort.^9^ Inclusion criteria were: (a) a diagnosis of probable or possible MSA;^10^ (b) an ENT examination in the voice and swallowing unit by an experienced ENT physician, within 100 days after or before the UMSARS assessment.

### Data Collection

Each patient was assessed by a standardized swallowing assessment, consisting of:

1. A nasofibroscopic (adopting the protocol described by Gandor and colleagues)^11^ and radioscopic exam (Data S1. for exam details). Swallowing disorders were characterized by:
  (a) clinical severity: (a) severe: massive primary and secondary inhalations, ineffective/absent cough, history or risk of pneumonia and presence of nutritional complications; (b) moderate: ineffective cough, possible inhalation, history or risk of pneumonia, moderate stasis, but no malnutrition; (c) mild: no primary and secondary inhalations, slight complications. Stasis of saliva or alimentary product has been classified as mild, moderate, and severe, in agreement with the physician's subjective judgment after product intake;
  (b) presence (yes/no) of choking/laryngeal penetration, corresponding to the visualization of food or liquids passing into the larynx;
  (c) presence (yes/no) of an airway protective mechanism (closure of the vocal cords, effective cough);
  (d) the presence (yes/no) of an oral transit defect, most often coinciding with retention of food in the mouth due to lack of initiation of swallowing;
  (e) presence (yes/no) of a pharyngeal transit defect resulting in the presence of food residue in the valleculae and/or the pyriform sinuses after the effort of swallowing;
2. Collection of pulmonary events (pneumonia) since disease onset and current nutritional complications (including at least one of the three following conditions: malnutrition, defined as a body max index [BMI] < 21, changes in food consistency and need of nutritional supplement).
3. The Deglutition Handicap Index (DHI) score, a self‐administered questionnaire on quality of life (QoL) related to swallowing problems.^12^

### Statistical Analysis

See Data S1.

## Results

### Clinical Features

From the 301 screened MSA patients, 101 had an ENT assessment, of whom the 75 with an interval less than 100 days between the ENT and UMSARS assessment were included. Demographic and clinical data are detailed in Table 1.

**TABLE 1:** Demographic, Clinical and Therapeutic Data of the Patients

Clinical data	n = 75
Female, %	58.6%
Age at diagnosis in years, mean (SD)	63.5 (7.6)
Diagnosis, n (%)	
Probable MSA	72 (96.0)
Possible MSA	3 (4.0)
MSA‐P	55 (70.7)
MSA‐C	22 (29.7)
Age at ENT assessment, mean (SD)	63.6 (8)
Disease duration at ENT assessment, mean (SD)	4.2 (2.4)
Time between first symptoms and death, mean (SD)	8.6 (3.2)
Age at death, mean (SD)	68.2 (7.7)
Death, number (%)	50 (66%)
Patients on levodopa, n (%)	60 (80)
Levodopa dose in mg, mean (SD)	671.4 (354.6)
Patients with at least one nutritional complication^a^, n (%)	49 (69.1)
BMI < 21, n (%)	26 (34.6)
Changes in food consistency^a^ n (%)	40 (56.3)
Nutritional supplement, n (%)	17 (22.6)
Pulmonary complications^b^, n (%)	20 (30.3)
Interval between ENT and UMSARS in days, mean (SD)	36 (27.8)
UMSARS part I score, mean (SD)	34 (18.5)
UMSARS part II score, mean (SD)	30.2 (8.8)
UMSARS part I (item 2, swallowing), n (%)	
0 – Normal	0
1 – Mild impairment. Choking less than once a week	18 (24)
2 – Moderate impairment. Occasional food aspiration with choking more than once a week	35 (46.7)
3 – Marked impairment. Frequent food aspiration	17 (22.7)
4 – Nasogastric tube or gastrostomy feeding	5 (6.6)
UMSARS‐ IV, n (%)	
1	0
2	13 (17.3)
3	9 (12)
4	41 (54.7)
5	12 (16)
DHI, mean (SD)^c^	38 (1.1)

^a^ 4 missing values.

^b^ 9 missing values.

^c^ Data available for 55 patients.

Abbreviations: DHI, Deglutition Handicap Index; ENT, ear, nose, and throat.

According to the UMSARS part I item 2 score, patients were classified as follows: 18 (24%) scored 1, 35 (46.6%) scored 2, 17 (22.6%) scored 3 and 5 (0.6%) scored 4 (Table 1). Pharyngo‐laryngeal signs related to swallowing are detailed in Table S1., indicating the presence of chocking and pharyngeal transit defects in about half of the patients (53.3% and 45.2%, respectively), and the absence of protective mechanisms in around one third of them.

### UMSARS‐Based versus ENT‐Based Dysphagia Assessment

**FIG. 1:**
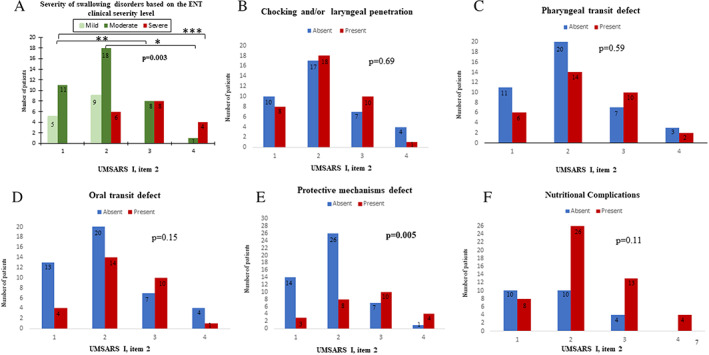
Distribution of the swallowing assessment features (ENT visit), stratified on UMSARS part I item 2 (swallowing) scores, ranging from: 0 – Normal, 1 – Mild impairment. Choking less than once a week, 2 – Moderate impairment. Occasional food aspiration with choking more than once a week, 3 – Marked impairment, frequent food aspiration to 4 – Nasogastric tube or gastrostomy feeding). On each bar the exact number of patients is reported. On the X‐axis of all the panel the UMSARS part I item 2 from 1 to 4. On the Y‐axis: (**A**) Number of the patients classified as mild, moderate and severe, based on ENT assessment (panel A; for 5 patients ENT severity assessment is missing). At pairwise comparisons, patients with a UMSARS part I item 2 score of 1 had less severe dysphagia at ENT assessment compared to those with a score of 3 or 4 (adjusted *P*: 0.012** and 0.005***). Patients with a UMSARS part I item 2 score of 2 had also less severe dysphagia at ENT assessment compared to those with score of 4 (adjusted *P*: 0.049*); (**B**) number of patients with/without choking and/or laryngeal penetrations (panel B); (**C**) (panel C); (**D**) number of patients with presence/absence of pharyngeal transit defect (panel D, for 2 patients missing data); (**E**) number of patients with presence/absence of oral transit defect (panel E, for 2 patients missing data); percentage of patients with presence/absence of at least one nutritional complication; (**F**) number of patients with presence/absence of protective mechanism (panel F).

The ENT assessment indicated more severe swallowing impairment compared to the UMSARS part I item 2 score (*P* = 0.003); patients scoring 2 at the UMSARS part I item 2 mostly had moderate dysphagia severity, patients scoring 3 were equally distributed among moderate and severe dysphagia while patients scoring 4 mostly had the worst dysphagia severity at the ENT observation (Fig. 1A). The absence/presence of choking was evenly distributed over the four levels of UMSARS dysphagia severity (*P* = 0.69) (Fig. 1B). Likewise, the distribution of an abnormal pharyngeal phase (Fig. 1C) or an oral transit defect (Fig. 1D) was not different across UMSARS part I item 2 severity scores (*P* = 0.59 and *P* = 0.15, respectively). Conversely, when analyzing the whole group, a significant difference in the distribution of the impairment of protective mechanisms across UMSARS part I item 2 scores was found (*P* = 0.005) (Fig. 1E), but with no statistical differences within UMSARS score groups at the pairwise comparisons.

### Dysphagia Complications and Related Quality of Life

The presence of previous pneumonia was not associated with the UMSARS part I item 2 score (*P* = 0.377). Likewise, the distribution of the occurrence of at least one nutritional complication was not significantly different over the UMSARS part I item 2 score level (*P* = 0.11) (Fig. 1F).

The DHI score tended to be more severe in MSA‐P (mean score of 35.6 ± 21 in MSA‐C and 46.2 ± 23.5 in MSA‐P; *P* = 0.067). The level of dysphagia related QoL reported by patients was well reflected by the global UMSARS part I item 2 score progression (p: 0.0001), with progressive worsening of QoL between scores 1 to 4 (global and two‐pair comparisons), except for a poor discrimination between the score of 2 versus 3 (*P* = 0.141) (Fig. S1.).

## Discussion

We performed a single center cross‐sectional retrospective study on 75 MSA patients, comparing the performances of the UMSARS part I item 2 versus the ENT assessment to grade the severity of dysphagia. The overall clinical severity of dysphagia and the defect of protective airway mechanisms follow the UMSARS I item 2 score progression. Indeed, the ENT assessment found “severe” dysphagia in 18%, 50% and 80% of the patients who had a UMSARS part I item 2 score of 2, 3 and 4, respectively. Likewise, the ENT assessment found an alteration of protective mechanism in 17.6%, 23.5%, 58.8% and 80% of the patients who had a UMSARS part I item 2 score of 1, 2, 3 and 4, respectively. However, not all aspects of the standardized swallowing assessment completely match with the UMSARS‐based evaluation. In particular, the presence of choking laryngeal penetrations did not follow the UMSARS item progression. Likewise, the presence of oral or pharyngeal transit defects was homogenously distributed over item progression. Conversely, the UMSARS‐based evaluation well mirrors the overall impact of swallowing problems on QoL.

The severity of swallowing disorders includes the concept of safety but also efficacy. Efficacy seems to not be addressed by the UMSARS part I item 2, as illustrated by the lack of a relation between the UMSARS severity level and the presence of an oral or pharyngeal phase abnormality. Indeed, the presence an oral or the pharyngeal phase abnormality increases until score 3 of the UMSARS scale, likely due to correlations established between the level of the pharyngeal/oral residue and the presence of choking,^13^ but it decreases at level 4 (only 3 patients available for the analysis).

Considering swallowing efficacy may allow improving the performance of a swallowing function assessment and identifying patients at risk for choking and nutritional complications. In this line, almost half of the patients scoring 2 or 3 at the UMSARS part I item 2 already presented impaired swallowing efficacy and a higher percentage of nutritional complications (69.1%). Moreover, severely affected patients do not necessarily have a UMSARS part 1 item 2 score of 4 because enteral nutrition may not have been started for reasons unrelated to the severity of dysphagia, including patient's refusal, clinical contraindication, and ethical considerations.

Increasing UMSARS part I item 2 scores seems not related to a higher percentage of chocking. This could be due to insidious/silent choking not disclosed during the patient interview (no cough and no throat clearing), as reported for Parkinson's disease.^14^, ^15^ Conversely, the defect of protective mechanisms, observed in about one third of the patients, is more prevalent among patients with higher UMSARS part I item 2 scores. The discrepancy among the prevalence of chocking and the one of protective mechanism defects, could indicate that the latter underlines a more severe swallowing impairment, but this finding would need to be replicated in a larger sample of MSA patients.

Main limitations of this study are the single center recruitment, the retrospective nature of the study and the bias related to the inclusion criteria. Indeed, only MSA patients referred for ENT assessment were included, thus with a risk of selection for patients with more severe dysphagia.

In conclusion, our study confirms that the UMSARS‐based assessment of dysphagia well reflects the overall severity of dysphagia but does not capture key aspects of swallowing efficiency related to pharyngo‐laryngeal function. According to the 2021 consensus statement on dysphagia management in MSA, dysphagia should be investigated through both available screening questionnaires, clinical (targeted on specific aspects) and instrumental assessment at the time of MSA diagnosis and periodically thereafter.^4^ Regarding the questionnaire‐based evaluation, the recently proposed MSA Swallowing Disturbance Questionnaire sub‐score may be a valuable screening tool, since it shows optimal correlation with dysphagia severity as detected on flexible endoscopic evaluation. On a related note, our findings suggest that the UMSARS swallowing item should be revised to better capture pharyngo‐laryngeal dysfunction in MSA patients, as part of the on‐going initiative to elaborate the new MDS‐UMSARS.^16^ We should also consider that it is likely that an instrumental assessment of dysphagia, at disease onset is not performed in all movement disorder centers. However, the early detection of these impairments by an ENT assessment may more efficiently allow the implementation of strategies to prevent malnutrition and aspiration pneumonia.

## Author Roles

(1) Research project: A. Conception, B. Organization, C. Execution; (2) Statistical Analysis: A. Design, B. Execution, C. Review and Critique; (3) Manuscript Preparation: A. Writing of the first draft, B. Review and Critique.

N.E.F.: 1A, 1B, 1C, 3A

A.P.T.: 1C, 2C

E.M.: 1C, 2C

O.R.: 1A, 3B

W.G.M.: 1A, 3B

A.F.‐S.: 1A, 2A

Y.G.: 1C, 2C

S.T.: 2A, 2B

F.O.‐M.: 1A, 2C, 3B

V.A.W.: 1A, 2C, 3B

M.F.: 1A, 2C, 3B.

## Disclosures

**Ethical Compliance Statement:** All patients provided written informed consent and the cohort is registered with the *Commission Nation ale Informatique et Liberté* (CNIL, no. 1,338,780; CCTIRS, no. 10.065). The authors confirm that we have read the Journal's position on issues involved in ethical publication and affirm that this work is consistent with those guidelines.

**Funding Sources and Conflicts of Interest:** This research did not receive any specific grant from funding agencies in the public, commercial, or not‐for‐profit sectors. The authors have no conflict of interest for this study.

**Financial Disclosures for the Previous 12 Months:** Nadia El Fassi reports no disclosure. Anne Pavy‐Le Traon reports honoraria from Biohaven. Emanuelle Mouchon reports no disclosure. Olivier Rascol has acted as a scientific advisor for drug companies developing antiparkinsonian medications (Abbott, Abbvie, Acorda, Adamas, BIAL, Biogen, Boehringer‐Ingelheim, Cynapsus, GSK, Impax, Merck, Osmotica, Oxford‐Biomedica, Lundbeck, Novartis, Prexton, Servier, Sunovion, TEVA, UCB, Zambon). Wassilios G. Meissner reports fees for editorial activities with Elsevier, consultancy fees from Lundbeck, Biohaven, Roche, Alterity, Servier, Inhibikase and Servier. Alexandra Foubert‐Saumier reports personal fees from LVL medical, outside the submitted work. Yohan Gallois reports no disclosure. Samuel Tessier reports no disclosure. Fabienne Ory‐Magne has received honoraria for serving as an advisory board member from Abbvie, Medtronic, Orphalan and Orkyn, and for consultancy activities from Aguettant, Abbvie, Orphalan, Homeperf and Orkyn. Virginie Woizard reports no disclosure. Margherita Fabbri received Honoraria to speak from AbbVie and BIAL.

## Supporting information


**Data S1.** Supporting Information

**Table S1.** Descriptive data of the pharyngo‐laryngeal signs concerning swallowing.
**Figure S1.** Correlation between the Deglutition Handicap Index (DHI) score and the UMSARS part I *item 2*. The DHI consists in 30 items, each one scoring from 0 to 4, with total score ranging from 0—no impact to 120—maximum impact; the scale is subdivided in three domains of ten items, ie physical [symptoms], functional [nutritional and respiratory consequences] and emotional [psychosocial consequences].
