# Supplementary material for: UMSARS Versus Laryngoscopy‐Based Assessment of Dysphagia
Source: Mov Disord Clin Pract. 2023 Apr 12;10(6):974–9. doi: 10.1002/mdc3.13734 (PMC10272892; doi:10.1002/mdc3.13734)
Supplement: Supplementary file 1 — Data S1. Supporting Information [file MDC3-10-974-s001.docx]

**Supplementary material**

*Methods*

*Radioscopic exam*

Radioscopic exam was performed as follows: the patient was seated and asked to intake a micropaque suspension thickened with a powder to obtain three consistencies: liquid, semi-liquid and thick. A 3cc volume was taken with a spoon, one for each consistency. The volume of the bolus was increased according to the course of the examination (stop if any chocking). The exam was video recorded, with 15 images/sec archived on an acquisition system. The following aspects were evaluated on the video: a) oral phase: bolus preparation, labial/velar continence, triggering and rocker propulsion; b) pharyngeal phase: cavum closure, nasal reflux, laryngeal ascension, pharyngeal propulsion, opening of the upper oesophageal sphincter and presence of residues in the vallecula and piriformis sinus; c) chocking presence and oesophageal phase.

*Statistical analysis*

Descriptive statistics of demographic and clinical data were provided for continuous (mean and standard deviation (SD)) and categorical (count and percentage) variables. Comparisons of the distribution of the UMSARS part I item 2 score severity (from 0 to 4) vs. the ENT clinical severity (mild, moderate, and severe) and vs. the presence of ENT-based swallowing disorders, nutritional complications, and previous pulmonary events were performed by means of Chi-squared test and Fisher’s exact test for qualitative variables, as appropriate. In case of a global statistically different distribution, pairwise comparisons were done for each UMSARS part I item 2 score group by means of the Fisher’s exact test, with Holms-Bonferroni correction applied to multiple comparisons.

Comparisons of DHI scores over the four UMSARS I item 2 groups, were done by means of the nonparametric Kruskal-Wallis tests, as values were not normally distributed. Then one to one comparisons were performed by means of the Mann Whitney test. The significance level was set at 95% (α = 0.05). The data were analysed using XLStat software, version 2021.2.2.
