# Supplementary material for: UMSARS Versus Laryngoscopy‐Based Assessment of Dysphagia
Source: Mov Disord Clin Pract. 2023 Apr 12;10(6):974–9. doi: 10.1002/mdc3.13734 (PMC10272892; doi:10.1002/mdc3.13734)
Supplement: Supplementary file 2 — Table S1. Descriptive data of the pharyngo‐laryngeal signs concerning swallowing. Figure S1. Correlation between the Deglutition Handicap Index (DHI) score and the UMSARS part I item 2. The DHI consists in 30 items, each one scoring from 0 to 4, with total score ranging from 0—no impact to 120—maximum impact; the scale is subdivided in three domains of ten items, ie physical [symptoms], functional [nutritional and respiratory consequences] and emotional [psychosocial consequences]. [file MDC3-10-974-s002.docx]

**Supplementary material**

**Table S1.** Descriptive data of the pharyngo-laryngeal signs concerning swallowing.

| **Variable** | **Missing value** | **Modality** | **number (%)** |
| --- | --- | --- | --- |
| **Swallowing impairment severity** | 5 | Mild  Moderate  Severe | 14 (20)  38 (54.2)  18 (25.8) |
| **Choking** | 0 | Present | 40 (53.3) |
| **Protective mechanism** | 2 | Absent | 24 (32.8) |
| **Oral transit defect** | 2 | Present | 27 (36.9) |
| **Pharyngeal transit defect** | 2 | Present | 33 (45.2) |
| **Pneumonia** | 9 | Present | 20 (30.3) |
| **Nutritional complications** | 9 | Present | 49 (69.1) |

**Figure S1.** Distribution of the DHI score based on the UMSARS part I item 2

UMSARS I – Item swallowing
